# Learning on the run – a qualitative, longitudinal study of pharmacy educators’ experiences implementing a hospital pharmacy residency program

**DOI:** 10.1186/s12909-022-03497-2

**Published:** 2022-06-04

**Authors:** Chih Yuan Wang, Alexandra Clavarino, Karl Winckel, Sonya Stacey, Karen Luetsch

**Affiliations:** 1 School of Pharmacy, The University of Queensland, 20 Cornwall Street, Woolloongabba, Queensland 4102 Australia; 2 School of Public Health, The University of Queensland, 288 Herston Road, Herston, Queensland 4006 Australia

**Keywords:** Pharmacy education, Pharmacy residency, Workplace training, Pharmacy educator

## Abstract

**Background:**

A hospital pharmacy foundation residency training program has been introduced in Australia, modelled on residency programs established in other countries. The program aims to support the professional development of early-career hospital pharmacists, in both clinical and non-clinical roles. Pharmacy educators are usually tasked with the implementation and maintenance of this program. This qualitative, longitudinal study aimed to investigate hospital pharmacy educators’ expectations, perceptions and experiences with implementing and developing their residency program.

**Methods:**

Qualitative data were collected at two timepoints, approximately 24 months apart, using either focus groups or interviews with pharmacy educators who were directly involved in the implementation of the residency program at their respective hospitals. During the early phases of implementation, and approximately 24 months later, participants were asked about their experiences and expectations of the residency program as well as any changes that had occurred within the residency program over time.

**Results:**

Four focus groups and three semi-structured interviews were held with pharmacy educators and senior pharmacists from different hospital settings. These were audio recorded and transcribed verbatim. Transcripts were inductively analysed via thematic analysis. Fifteen hospital pharmacy educators and senior hospital pharmacists participated in the initial focus groups and interviews, and seven educators were retained for follow-up. Four main themes were established from the discussions: participants had great expectations of a positive impact of the residency on their workplace and residents’ professional development; substantial effort, support and resources were needed to implement and maintain a residency program; self-motivation and engagement is needed by residents to succeed and experience timely completion and career acceleration; and lastly a balance between standardisation, consistency and flexibility in delivering the residency needs to be found. The role of educators changed with the implementation of a residency, with the addition of more managerial and supervisory aspects.

**Conclusion:**

The Australian hospital pharmacy foundation residency program is a complex workplace training program with multiple factors and prerequisites influencing its implementation, development and outcomes. Pharmacy educators are central to the successful implementation and ongoing sustainability of a residency program. They may benefit from formal training and qualifications to support their role.

**Supplementary Information:**

The online version contains supplementary material available at 10.1186/s12909-022-03497-2.

## Introduction

Over the last decade the number of registered pharmacists in Australia has increased by 36%, with at least 20% of pharmacists working in hospital settings [1–3]. In parallel, the roles of hospital pharmacists, like those of other healthcare professionals are becoming more varied, extended and specialised. Pharmacist involvement in patient care has evolved from a focus on medication supply to active participation in multidisciplinary health care and interprofessional practice. Pharmacists are now involved in optimising patient care, the ongoing management of medical conditions, for example, diabetes and asthma, as well as ensuring medication and patient safety [4–9].

Undergraduate and graduate entry pharmacy degrees may not adequately prepare pharmacy graduates for the increasing role diversity and complexity of contemporary healthcare, especially in hospital settings. Experiential and work-integrated learning, during undergraduate studies, often focuses on preparing graduates for community pharmacy practice as this is where the majority of the pharmacy workforce is based [3].

To support and maintain the professional development of hospital pharmacists in the early phases of their career various strategies, including structured residency and other workplace-based training programs, have been developed, particularly in the United States of America and the United Kingdom [10–13]. Since late 2016 a training program led and designed by the Society of Hospital Pharmacists of Australia (SHPA), has been progressively implemented in Australian hospitals, modelled on pharmacy residency programs in these countries [14–17]. The SHPA Foundation Residency program (referred to as ‘residency’ from hereon) establishes a framework for the introduction of a formal, structured, and nationally accredited two-year workplace training program for early-career and new-to-hospital pharmacists. It offers early-career hospital pharmacists the opportunity of guided professional development in both clinical and non-clinical roles after completing their compulsory one-year internship and board registration [16–18]. Recognising the need for early-career pharmacists to build a more solid foundation in hospital pharmacy practice, the residency program aims to provide a bridge between the pre-registration intern training program and the first stage of the three advanced competency levels of the National Competency Standards Framework for Pharmacists in Australia 2016 [16, 18]. Although currently suspended, an option for residents and other pharmacists to pursue advanced practice credentialling has been in place in the past [19].

This two-year program consists of three six-months rotations in medical, surgical and operational areas, as well as one six-month elective in an area of interest. During their residency, resident pharmacists (‘residents’ from hereon) are required to complete multiple clinical or practice-based and non-clinical assessments and tasks. The clinical and practiced-based assessments are described in Table 1, the non-clinical tasks for the entire residency include case presentations and discussions, reflective logs, research projects with manuscript preparation, compulsory educational seminar attendances, teaching of students and participation in higher duties such as attending working groups and committees. These activities are all supervised by pharmacy educators or other senior pharmacists, with educators usually organising and conducting clinical assessments and rotations [16–18, 20, 26]. While this may vary between individual hospital pharmacies, there is no formal obligation for residents to stay on as staff pharmacists after completion of the residency program.

**Table 1:** Description of assessments and other requirements to complete a residency

Assessment	Description
One per rotation SHPA Clinical Competency Assessment Tool (ClinCAT) Evaluation	The ClinCAT is a peer review of competencies, evaluating clinical and professional skills of a resident via direct observation. It involves both self-assessment by the resident and observation by the evaluator. The result of the ClinCAT is discussed by the two parties in order to form an agreed action plan. The time to undertake a ClinCAT usually requires 4 h from both the pharmacist and the evaluator. The evaluator needs to complete training and register with SHPA as an accredited ClinCAT evaluator [20, 21].
Monthly Mini-Clinical Evaluation Exercise (mini-CEX)	The mini-CEX is a short and targeted evaluation tool to evaluate and facilitate feedback on clinical and critical thinking skills, attitudes and behaviours in a specific practice area. The time to undertake a mini-CEX usually requires 15–30 minutes for both the resident and evaluator. The evaluator does not require specific accreditation and can be a senior pharmacist, mentor or educator at the workplace [20, 22].
One per rotation Mini-Peer Assessment Tool (mini-PAT)	The mini-PAT is a peer assessment tool which facilitates peer feedback on the resident’s professional performance, skills, attitude and behaviours. The evaluatee conducts a self-evaluation and a range of selected peers who work with the resident provide feedback against a set of criteria. The collated feedback with the self-evaluation is reviewed and discussed by the resident and the educator or mentor to form an agreed action plan for further skill development [20, 23].
Seminar requirements	In the early implementation phase of the residency, residents were required to attend two SHPA seminars in clinical medication management. This has been changed to attendance of one SHPA seminar and one other continuing professional development event of the resident’s choice. Residents needs to submit the program outline and reflection on their learnings to demonstrate the suitability of the non-SHPA seminar [20, 24, 25].

Participation by hospital pharmacies in the residency is voluntary, but an individual site must submit an application to SHPA demonstrating their capabilities in education, training, research, and prove they have the necessary organisational resources to become a provisionally or fully accredited residency sites [27]. Hospital pharmacy sites must also designate a pharmacy educator and residency program lead to oversee and manage the program [27]. The role of program lead is typically taken on by a director or assistant director of pharmacy, in some circumstances the pharmacy educator may also fill this role.

The role of a hospital pharmacy educator (‘educator’ from hereon) and their scope of practice varies between different hospitals. Educators in Australia typically have supervisory roles as preceptors for intern pharmacists and students in their organisation, they often assess clinical assessments and conduct, organise or manage departmental education events [28, 29]. The introduction and implementation of the residency has affected educators’ everyday duties by adding new responsibilities to their role. These include managing and facilitating the formal training program, ensuring that all mandated training opportunities are provided and assessment requirements are met by resident pharmacists, all of which can be resource intensive and supplementary to workplaces’ usual training pathways.

For this study, we defined hospital pharmacy educators as pharmacists who have the designated role of overseeing education at their hospital site, this may be in either a full- or part-time capacity, and oversee the educational aspects of residency programs. For example, in some smaller hospitals senior pharmacists take on a part-time educator role, whereas bigger, tertiary hospitals often have the resources to employ educators in a more permanent and full-time capacity.

### Aim

This qualitative, longitudinal study aimed to investigate educators’ expectations, perceptions and experiences with implementing and developing their residency program over time.

Findings may inform future implementation of and adjustments to existing residency programs and identify potential support needed by pharmacy educators.

### Ethics approval

This study was approved by The University of Queensland, Human Research Ethics Committees (Approvals: 2017000827). All participants provided written, informed consent before voluntarily participating in this study.

## Methods

Reporting of this study follows the Standards for Reporting Qualitative Research Checklist (Additional file 1) [30].

### Participants and recruitment

Educators and senior pharmacists with an education responsibility, who self-identified as the facilitator, or had a significant involvement in the implementation or management of the training aspects of residencies at their hospital, were invited to participate. Participants were identified and invited either through the study investigators’ professional and personal networks, publicly available SHPA data or opportunistically at professional meetings.

### Data collection

Face-to-face and online focus groups or semi-structured interviews were conducted. Online focus groups were conducted via video conferencing software (Zoom Video Communications Inc.). Semi-structured interviews were conducted via phone or Zoom when participants were unable to attend in person due to geographical location, scheduling or social distancing requirements. While there are both advantages and disadvantages for participation in focus group or semi-structred interview, the investigators ensured that all participants were provided opportunities to express their views freely regardless of the mode. Utilising both modes also improved the attendance of participants and ensured reach to a more diverse group of educators [31]. The primary investigator (CW) conducted all focus group discussions with one other co-investigator (KL, AC) who had extensive focus-group and small group facilitation skills. Prompting questions were used to start discussions, investigators then facilitated rather than guided the discussions to provide an opportunity to educators to determine the direction and to ensure equal contribution from all participants. No incentives to participate were offered.

All focus group and interviews were audio recorded and transcribed verbatim by either professional transcription services or the main investigator (CW), who also checked the accuracy of professionally transcribed transcripts.

The baseline data collection started in late 2018, continuing into 2019 to capture late initiators of residency, and to ensure most participants had experience in implementing or managing their residency for a year. Transcripts of both focus groups and interviews were reviewed iteratively and data collection ended when this review showed that participants did not offer new insights and answers to interview questions and it was anticipated that no new themes were able to be generated from the data [32]. Follow up data was collected at one point in time in 2021, this was approximately 24 months after the completion of the initial sessions. The aim of this follow-up was to explore whether any changes in educators’ experiences or the the residency programme occurred over time.

### Data analysis

As the questions were the same for focus groups and interviews, transcripts from each round of data collection were combined for the data analysis. Microsoft Excel was used to sort and categorise data. An inductive data-driven approach to thematic analysis of the transcripts was followed, where all investigators reviewed the transcripts and independently developed preliminary themes. Main themes were established via frequent group discussion, after which three investigators (CW, KL, AC) established codes for all themes and then independently coded all transcripts [31, 33]. Regular meetings were held to resolve any inter-coder differences and to establish the final coding frame and refinement of themes.

## Findings

Over the course of the study four focus groups and three semi-structured interviews were conducted. Two focus groups and two semi-structured interviews were undertaken at the first stage of data collection, two focus groups and one semi-structured interview collected follow-up data. On average, each focus group lasted approximately 60 minutes and interviews were approximately 40 minutes in duration. The discussions encouraged a wide range of responses, participants shared their experiences and views freely. Overall, 15 stakeholders from 10 different hospital sites, and different hospital settings, participated. The participants were recruited from three different States in Australia. They were representing one third of all the early adopting sites of the Australian program. The majority of respondents represented hospitals from the one State which during the early phases of implementation in 2017 had the highest uptake of residency. All partipants were pharmacy educators, working either in a full- or part-time capacity, while some may also have been a designated program lead for periods of time, their substantial role, as they described it, was in education. All participants were involved in the implementation and introduction of residency programs at their hospital or health service and identified as the person with the most significant educational role in their local residency programs.

Most educators (*n* = 13) worked in tertiary hospitals (Table 2). Seven educators from the initial focus groups and interviews participated in the follow up sessions; many of the original participants were no longer in the role of educator in 2021.

**Table 2:** Participant demographic data

Participant	Session of focus group/interviews	Category	Gender	Age group (years)	Current site of employment (secondary or tertiary hospital)	Current site offers residency program
E1	FG1 & I3	E	F	51–60	Tertiary	Yes
E2	FG1 & FG3	E	F	31–40	Tertiary	Yes
E3	FG1 & FG3	E	F	41–50	Secondary	Yes
E4	FG1	SP	F	31–40	Tertiary	Yes
E5^a^	FG1	SP	F	41–50	Secondary	No
E6	FG2	SP	F	21–30	Tertiary	Yes
E7	FG2 & FG4	E	F	41–50	Tertiary	Yes
E8	FG2	E	M	41–50	Tertiary	Yes
E9	FG2	E	F	21–30	Tertiary	Yes
E10	FG2	E	F	31–40	Tertiary	Yes
E11	FG2	E	F	31–40	Tertiary	Yes
E12	I1 & FG4	E	F	31–40	Tertiary	Yes
E13	I1 & FG4	E	F	31–40	Tertiary	Yes
E14	I2 & FG4	E	F	21–30	Tertiary	Yes
E15	FG3	E	M	31–40	Tertiary	Yes

*FG* Focus group, *I* Interview, *E* Educator, *SP* Senior Pharmacist with educator role, *F* Female, *M* Male

^a^Participant E5 was included as part of the initial data collection as they were preparing to launch their residency, however, their site was not accredited at the time of data collection

Four main themes were identified. These related to educators’ expectations; effort, support and resources needed to implement and maintain a residency; motivation and engagement needed for program success; and the balance between standardisation and flexibility of the program. Quotes illustrating each theme are presented in text and Tables 3, 4 and 5.

**Table 3:** Theme 2

Theme 2: It takes a village to raise a village - effort, support, and resources needed	
Subthemes	Example of quotes from transcripts
2.1 Resource requirements – workload constraints	It’s not just the educator; it’s not just a single person raising the village. It’s up to everybody to be engaged in this. *E5, FG1* I think it’s been an increase in workload and responsibility, which is both good and bad for everybody, not just the residents and the educators, but the team leaders as well… *E10, FG2* …. I think the team leaders initially, they were often the mentors, and they were over-committed, whereas now, we’ve got mentors who are often people who’ve done the residency before or not necessarily a team leader, and I think that’s really been good. *E1, I3*
2.2 Resources requirements – skill sets required	It has been a step up for the clinical educators but it’s also been a huge step up for middle management, I suppose, and people who have been in the system for say 20 years who have managed, this is quite a new concept. There are people before who never had to give feedback, haven’t had to use some of the tools, and now all of a sudden, they need to do all those tasks. *E10, FG2* What I’m finding is probably the change over the years is that initially we had very limited support when it came to our senior pharmacists actually having that experience with research to then support residents. *E7, FG4* … the university have partnered with us, and we basically do like a panel review of their research early on, ... all that support that’s sort of needed to have a good quality project at the end… and that seems to have been a really big winner for our residents. *E13, FG4* … with the capacity to actually train, probably the biggest evolution is our graduate residents. The most effective trainers of the residency that I have on my staff, pretty much, because they know the program. So my capacity to do the training program has increased. *E15, FG3*

**Table 4:** Theme 3

Theme 3. You can’t fake it, but you can still make it – need for motivation and engagement	
Subthemes	Example of quotes from transcripts
3.1 Motivation of the residents	We have to deliver the programs (*residency*) but I think it’s more the residents. You can feel - I have seen the growth of the residents that have really taken it and run with it, whereas the ones that haven’t, they still just - it’s just like a general kind of thing and I just feel like the ones that have embraced it grow. And it’s good to see because you can reflect and see how they grow. *E3, FG1* … obviously it depends on their motivation. So it’s not to say that for everyone who goes through the residency program, the outcome will be the same, because it’s the intrinsic factors which play a big part as well. But the structure definitely helps. *E12, FG4*
3.2 Alignment of expectations	… we have a sit-down at the start and sort of outline what the expectations are. And what we’ve learned over the time is they do have to be engaged with it, and commit to the requirements, otherwise it’s just a hard slog for everybody, and you really don’t achieve what you’re setting out to achieve. So we outline it that way... *E2, FG3* We put that *(responsibility)* back onto the resident, and we’ve had to clarify, make the expectations much clearer, because that was an issue at the start, and just people doing different things across the sites... So it’s trying to sort of marry up those expectations. *E12, FG4*

**Table 5:** Theme 4

Theme 4: Not one size fits all – standardisation versus flexibility	
Subthemes	Example of quotes from transcripts
4.1 Adjustments to the residency	... I think at this early-stage flexibility is really important. In five or 10 years’ time, you can start lifting the quality but I think, at the moment, if some sites don’t have the capacity to do all of these things for all of their people that want to be residents, I think they should recognise that they are trying too quickly to make it high quality without getting buy in from the base. *E8, FG2* We have kind of tailored our frequency right from the start. And SHPA was happy with that. We did need to tweak a few things. *E12, FG4*
4.2 Program design	… Some of it (*residency activities and tasks*) is not being built really thinking about curriculum and learning needs and all that sort of stuff. Some of it is being built to fix the department’s problems rather than development of the residents… *E8, FG2* So we have been trying to turn it around so that we actually focus on them attending seminars that target their learning needs and their gaps which they can identify themselves through all the feedback and assessments that we do. *E10, FG2*
4.3 Concern about flexibility leading to inconsistencies	… it was super-flexible at the start because no-one knew what they were doing. *E8, FG2* We have got such a mixed implementation of our residencies through the different sites. It means something different per site on what it looks like with regards to permanency, temporary positions, what you get at the end, if you get anything at the end... *E5, FG1* I was thinking about consistency between sites: Is this resident from the “Hospital A” the same as “Hospital B”, the same as “Hospital C”? I don’t think we have really established that yet. So accreditation for five versus 3 years, well, your program might change after that, yet these guys are still doing it. How can you for employment say that this resident matches this resident? … *E9, FG2*

### Theme 1. Great expectations – the initial phase and future of residency

From the start educators had high expectations of what the implementation of a residency would achieve for their workplace and residents, althoughthe uptake into the residency program differed across sites. The selection process of residents was not standardised, each site was responsible for their own recruitment. Larger, more well resourced tertiary hospitals were at times able to implement an opt-out approach, offering participation in the residency to all their early career pharmacists. Most hospitals, however, adopted an opt-in approach and a competitive, merit-based selection process, which resulted in ‘top of class’ early-career pharmacists joining their residency.

‘I have an expectation that the residency will meet the development needs of early-career pharmacist. But what we are doing at the moment is, we are attracting the highest quality sort of early-career pharmacist to be involved in the program. I would like to see this available to those who struggle a bit more and maybe don’t fit in that top cohort.’ *E13, I1*

Educators thought that residents also had high expectations of their achievements.

‘They (*residents*) see themselves more superior. They are fully aware of the timeframe, and they don’t want to be seen as not complying with the program requirements. Yes, ours are probably less comfortable with failing, the idea of failing. I would like to hear what they actually say but they definitely have this feeling of superiority, I can tell you.’ *E4, FG1*

Across the period between initial interviews as well as follow-up, educators continued to remain concerned that the demands of the residency and the selection process would inadvertently create a two-tiered system that favoured training opportunities for residents over non-residents. Whilst individual hospital pharmacies may have had well-established training programs for non-residents, workplaces inadvertently had to prioritise potentially limited training resources and opportunities for their residents in order to ensure they met residency and accreditation requirements. The selection of “top of class” pharmacists also seemed to contribute to the development of the two-tiered system.

‘… It's still an issue, and as much as I've tried to combat that with every resource I can think of, but it's a little bit of the elitism, a bit of the golden child. … They (*the residents*) get the best rotations.’ *E15, FG3*

In the follow-up interviews all educators agreed that despite the challenges, which are also described in the following three themes, the implementation of the residency was highly beneficial to their workplace and all pharmacists participating in it.

### Theme 2. It takes a village to raise a village - effort, support, and resources needed

At the initial interviews educators emphasised that significant time, resources and adjustments were needed in workplaces as well as high levels of individual commitment by residents, preceptors and mentors to implement and maintain a residency. The success and sustainability of the residency required commitment from all pharmacy staff, not just educators who led the implementation, as the additional workload created by the implementation and ongoing management affected everyone. Given the time intensive nature of some of the frequently performed competency assessments (see Table 1), additional time was required by educators and pharmacists overseeing rotations to mentor and assess residents in line with SHPA requirements. From the educators’ point of view, this created pressure for assessors and residents, particularly if the assessors were also tasked with conducting assessments for other, non-resident pharmacists in the process of regular workplace training and performance development. Educators also took on additional managerial responsibilities, e.g. organising rotations and mentors, reconciling residents’ needs with operational needs and supervising residents’ overall progress (subtheme 2.1).

Educators and other supervisors reported that the skill sets necessary to supervise and assess residents were not always readily available in their workplaces. They commented on shortfalls in the number of pharmacists with adequate educational or mentoring skills as well as the clinical experience required to conduct clinical assessments and provide feedback to residents, with only few pharmacists available to act as suitable mentors and assessors (subtheme 2.2). A potential lack of research skills by those supervising residents in their quality assurance or research projects was also highlighted, this created challenges in providing sufficient support or guidance to residents.

These initial challenges changed significantly over time. At the follow up focus-groups and interviews, educators described the various strategies they used to resolve or mitigate these issues. Within one of the more centralised hospital health services, the residency was implemented using a hub and spoke model, with residents rotating through various hospitals that were able to offer specific rotations that were not necessarily available within one individual hospital. The residency program was also aligned with a local university-based post-graduate program. Collaboration with a university added educational capacity to the residency program, with university academic staff conducting some practice-based assessments in the workplace and supervising residents’ research projects. These strategies resolved potential issues associated with time constraints and skill deficits in workplaces.

All educators experienced greater engagement from all staff over time, with mentoring becoming more normalised for senior clinical staff. Importantly, pharmacists who had completed the residency program and remained on staff supported new residents as mentors and program experts. They actively committed to assisting educators in maintaining their residency program by conducting assessments and supervising research projects. For many educators these developments made the initial work and resources they had to direct to the residency worthwhile.

### Theme 3. You can’t fake it, but you can still make it – need for motivation and engagement

While the residency itself provided a sound structure for training, outcomes were highly dependent on each individual resident’s engagement with the program.

‘...I do feel that (*residency*) does give them that opportunity but… it just is how they use those opportunities.’ *E4, FG1*

Educators found that if residents did not engage with the program and made limited effort, their skills, behaviours and attitudes could remain unchanged at the end of the two-year training despite educators’ input and efforts (subtheme 3.1). Outcomes were perceived to depend on how the resident participated in the program.

‘I think that somebody could be spat out at the other end with no change to them at all, because we see that with standard rotations ... We can see those (*residents*) that just want to rock up... it’s not a guarantee that, if you are in the same program, that you will all come out the same’. *E5, FG1*

Educators also learnt to elicit and align everyone’s expectations of the residency. Some residents had unrealistic expectations about the level of commitment needed to successfully complete their residency. In the early stages of implementation mismatches in expectations of responsibilities between residents and educators often lead to unsatisfactory experiences for both parties, and in some instances discontinuation of a resident’s training (subtheme 3.2).

The educators believed that having an early exposure to a variety of potential pharmacy career pathways, inherent to the structure of the residency, was important for the residents’ future career development as it allowed them to identify their personal learning interests and potential career goals. Residency potentially shortened the time residents needed to explore their preferred career pathways.

‘You may not excel at that but then you will know that is not my cup of tea…’ *E3, FG1*

When residents engaged, rotations, supervision of students, quality assurance and research projects provided experiences that exposed them to a wide variety of roles, giving them authority and opportunities to take on roles they may not have been granted otherwise early in their careers. Even when not all activities were completed successfully, educators still thought the different experiences provided valuable learning opportunities and often observed that these benefited residents’ career development and acceleration.

‘… we've had people move on to (*senior pharmacist*), permanent (*senior pharmacist*) positions. We were devastated when they left, but yeah, so I think it just equips people to a level where they're just ready to get onto, to take on, well, they all, their practice is at an (*senior pharmacist*) level.’ *E3, FG3*

### Theme 4. Not one size fits all – standardisation versus flexibility of the residency

Although the residency program was initially designed as a nationally standardised and accredited program, all educators made adjustments in order to implement or maintain their local residency (subtheme 4.1). Adjustments were more often driven by workplace needs and capacity rather than by residents’ needs. One commonly reported adjustment overt time was a reduction in the frequency of the resource-intensive, practiced-based assessments as these were found to be time consuming and staff with appropriate skillsets required was not always available.

Reconciling workplace needs, SHPA standards and residents’ development needs remained challenging for educators. Educators confirmed that some positive changes occurred based on feedback by educators to SHPA, for example by giving residents the freedom to choose one of the two compulsory seminars according to their learning needs and interests. From the educators’ perspective some of the program activities lacked clear learning and training objectives, which raised questions about the purpose of some of the residency activities and requirements (subtheme 4.2).

While some flexibility was needed to allow each residency site to cope with the program requirements, the educators were concerned that this flexibility had led to significant program variation between different sites, which may, over time, affect the credibility, validity and cohesiveness of the program and importantly, the quality of residents’ experiences (subtheme 4.3).

Educators also emphasised the need for SHPA to evaluate and provide further guidance on reviewing and developing the residency curriculum.

‘… it has been two years now, and it’s time, they maybe did have feedback sessions or groups like these, maybe to see what program leaders and residents are thinking about the program and what could be done better.’ *E7*, *FG2*

## Discussion

This qualitative study of pharmacy educators’ experiences with the SHPA foundation residency in Australia elicited many of the challenges educators faced in the early phases of program and in some cases continue to face up to 2 years later.

The study described the strategies some educators had used to overcome these challenges, and pointed to changes in the educator role throughout the implementation and maintenance of the program. Currently, literature investigating pharmacy educators’ experiences implementing a residency at a national level is limited. Most studies reported on the experience in single healthcare services and longitudinal observations on how experiences change over time are lacking [34–36]. They reported on localised challenges, e.g. sustaining funding for resident and post residency positions, communication between supervisors to ensure a clear understanding of residents’ progress and the development of time management skills by residents [34–36]. In comparison, this study provides unique insights from educators over a longer period of time regarding a nationwide program.

Educators’ experiences showed that the SHPA’s intended aim of hospital pharmacy workforce development via a residency program is achievable, and can facilitate the career transition of junior pharmacists from internship to more advanced practice. Educators described the residency as a valuable, but resource-intensive, workplace training program, that to succeed required skilled educators and other senior pharmacists with the time to devote to the program. Their experiences showed that drawing on locally available skills, by either keeping graduated residents on staff, or collaborating with other hospital pharmacies or universities, mitigated potential resource limitations and skill shortages. The extension of residency implementation from tertiary hospitals to smaller, usually less well resourced, secondary hospitals could be facilitated by exchanging residents for rotations and having senior pharmacists or academics from tertiary hospitals or universities provide additional educational support. Engaging additional experience and expertise to support residents in more complex areas, such as research, either from other hospital departments or externally, would also enhance the residents’ learning and supervisory experience. Aligning the program with postgraduate study could further distribute workload and assessment requirements throughout the residency. This would allow for a broader implementation of residency programs across Australian hospitals, at the same time raising the skill level of a greater proportion of the hospital pharmacy workforce and avoiding concerns of selectivity and exclusivity educators raised.

Educators described having a pivotal role and influence in the residency, which supports the SHPA stipulation of a designated educator at a site as a program requirement. While the educator role has always included the supervision of students and interns, developing and organising site specific training, and supervision and educational events, with the implementation of a residency program their role has been extended. Taking responsibility for the implementation of a structured workplace training program, which prescribes a curriculum with regular assessments and formalised activities potentially demanded additional skills and expertise. In the early phases of residency uptake, educators were learning and exploring how to best implement and adjust the program to their workplace, developing their own strategies and skills in facilitating training of registered pharmacists. Changes they described to their role extended to the enlistment, support and supervision of senior pharmacists who were training and assessing residents, motivating residents, negotiating expectations and learning needs, and getting involved in curriculum development and program evaluation. These may become more challenging once residencies are offered more widely to all early-career pharmacists, including those who may be less motivated and engaged than current residents.

While SHPA outlined desirable competencies for pharmacy education almost a decade ago, there is limited consensus on how these may be achieved or what prerequisite qualifications would be useful [37–39]. Clinical pharmacy education is also not recognised as a formal speciality in the hospital pharmacy system of Australia, and proposed key skills have not been reviewed or updated in light of the residency program. Individual educators may obtain additional teaching qualifications or undertake mentorship or clinical assessment training offered by SHPA, however, educators do not necessarily have the experience and formal training for developing, facilitating or maintaining a structured workplace training program and its curriculum as hospital pharmacists often step into this role opportunistically.

In this study, about one-third of educators who participated in the initial discussions were no longer working in the education space 3 years later, suggesting that the educator role may be considered a short-term, temporary rotation in some hospitals.

Formal training and qualifications may allow educators to understand, critique and modify the residency program informed by adult and workplace-based learning theories, reconciling changes seemingly driven by organisational needs with residents’ learning needs.

New challenges posed by the implementation of residency, the addition of responsibilities of developing and adjusting the curriculum of a new workplace training program suggest that formal recognition of the role and skill of pharmacy educators in clinical and workplace settings could align more closely to the role of a medical educator, many of whom practice clinical education as a subspecialty of medical education, and it may be beneficial to create permanent, specialised pharmacy educator roles [40–42].

The experiences of medical and other health professional educators’ show that they take on more responsibilities than simply sharing clinical knowledge and skills as a clinical supervisor [41]. Educators in other health professions, who are involved in workplace training, have additional roles in teaching, research and management [43]. These skills can be acquired through specialised training, e.g. postgraduate certificates and courses in medical or clinical education [42, 44, 45]. In addition to professional content knowledge, educators need to have skills and knowledge in curriculum development and program design for the programs they facilitate, and have the abilities to implement, facilitate and manage them [41, 46–50]. Educators are also expected to be educational leaders, mentors and innovators in their workplace, who engage in researching, evaluating, and improving the education experiences for learners and ensure optimum learning outcomes [41, 46, 48, 50]. Knowledge of educational theories, pedagogy, and contemporary teaching strategies supports educators in being effective, facilitating adult learning for health professionals in workplace training programs [43, 51–56].

Educators flagged the need for evaluation and modification of the residency, as it became evident that individual programs have been evolving independently. Variations between residency sites, while necessary at times, jeopardise the benefits that a standardised workforce development program promises. Regular evaluations of the consistency of program implementation and reviews of contemporary learning needs of early-career pharmacists could lead to evidence-informed adjustments to ensure sustainability and wider uptake of the residency.

### Limitations

This study was a preliminary qualitative study of pharmacy educators involved in the implementation and development of the initial roll-out of the pharmacy residency program. It did not take into account the views of other key stakeholders, including management and the residents themselves, which are explored separately. Participants were from different States and practice settings in Australia, however, a significant proportion of participants were drawn from one State, because the residency program was initiated there earlier than in others. Participants primarily worked in tertiary hospital settings. This was expected as tertiary hospitals are often better resourced to implement new programs, and thus may be more willing to trial a new training program early compared to smaller or rural hospital pharmacies [57]. Hence, findings from this study may not be generalisable to all hospital pharmacies in Australia or internationally. Furthermore, half of the original participants were lost to follow-up but may have had relevant information regarding residency to share at their local sites.

## Conclusion

Pharmacy educators described the residency as a useful program to accelerate professional development but also as a complex workplace training program with multiple factors influencing its development and outcomes. Educators are central to the successful implementation and ongoing sustainability of a residency program and may benefit from formal training and qualifications to support their role and the ongoing evaluation and modification of the residency program across Australia. As the hospital pharmacy workforce expands, clinical pharmacy education could be established as a speciality as are the clinical aspects of medical education, with similar prerequisite training as is required for roles in other pharmacy specialty areas.

## Supplementary Information

**Additional file 1.**

## Data Availability

The datasets generated and/or analysed during the current study are not publicly available due to privacy or ethical restrictions but are available from the corresponding author on reasonable request.
